# Integrative Multi‐Omics Approaches to improve early Detection and Decipher Causal Pathways in Dementia

**DOI:** 10.1002/alz70860_103099

**Published:** 2025-12-23

**Authors:** Petroula Proitsi

**Affiliations:** ^1^ Centre for Preventive Neurology, Wolfson Institute of Population Health, Queen Mary University of London, London, United Kingdom

## Abstract

**Background:**

Developing effective strategies to prevent, delay, or halt the development of dementia is challenging due to its complex etiology and long preclinical phase. There is an urgent need to identify accessible biomarkers to better detect individuals at risk of developing dementia as early as possible and to understand the causes of dementia. We integrated genetic, multi‐omic, and modifiable risk factor data to improve early dementia detection and unravel causal pathways in dementia.

**Method:**

Using data from the UK Biobank, we investigated associations between blood proteins (Olink) and metabolites (Nightingale Health) with incident dementia over a follow‐up period of up to 15 years. Cox Proportional Hazard Lasso models were used to identify Metabolite and Protein signatures predictive of all‐cause dementia, Alzheimer's disease, and vascular dementia. We examined the associations of these signatures and key molecules with endophenotypes including regional brain volumes, to gain insights into early preclinical dementia and the underlying pathophysiology. To uncover causal relationships, we employed Mendelian randomization (MR) and statistical colocalization using the largest genome‐wide association studies for proteins, metabolites, gene expression levels, dementia, and endophenotype outcomes. We used formal causal mediation analyses (Multivariable MR) to investigate the interplay between molecules, modifiable risk factors, and dementia, to identify potential targetable molecular pathways. Analyses were stratified by biological sex, and evidence was triangulated from observational and causal inference approaches.

**Result:**

We identified multi‐omics signatures with shared and distinct molecular drivers influencing different forms of dementia. Our analyses revealed both APOE‐ε4‐dependent and independent associations with dementia risk, alongside novel links between molecules and brain volumes. MR analyses suggested that the associations between key metabolic and protein markers with dementia outcomes and brain volumes may be causal. Specific proteins involved in immune processes and lipids mediated the relationship between cardiometabolic risk factors—such as diabetes and obesity—and dementia. Some associations were more pronounced in women.

**Conclusions:**

Our findings highlight the power of integrating multi‐omics data using machine learning and causal inference to enhance early detection and elucidate causal pathways in dementia. We identified key molecules as promising candidates for preventive and therapeutic interventions, aiding personalized medicine and ultimately reducing the burden of dementia.

